# Supplementary material for: Adaptation of eukaryotic membrane homeostasis to species-specific cellular lipid landscapes
Source: Genes Dev. 2026 Sep 1;40(17-18):1429–47. doi: 10.1101/gad.353500.125 (PMC13532661; doi:10.1101/gad.353500.125)
Supplement: Supplementary file 1 [file Supplemental_Information.pdf]

Adaptation of eukaryotic membrane homeostasis to species-specific cellular lipid landscapes

Supplemental Information

Supplemental Figures

Supplemental Figure S1. The loss of Mga2 results in decreased fatty acid unsaturation and further remodelling of the cellular lipidome in both fission yeasts.

Supplemental Figure S2. Mga2 is proteolytically activated via the proteasome in both sister species.

Supplemental Figure S3. Mga2 transcriptional activator domain is functionally interchangeable between *S. pombe* and *S. japonicus*.

Supplemental Figure S4. Experiments defining the region responsible for the species-specific Mga2 cleavage patterns in fission yeast.

Supplemental Figure S5. *S. pombe ole1* intergenic region shows major rearrangements.

Supplemental Files

Supplemental Data 1. Lipidomics. All data presented as Excel tables.

Supplemental Data 2. mRNA-seq.

Supplemental Data 3. *S. pombe* Mga2 variants.

Supplemental Data 4. List of fission yeast strains, primers and plasmids used in this study.

Supplemental Figures

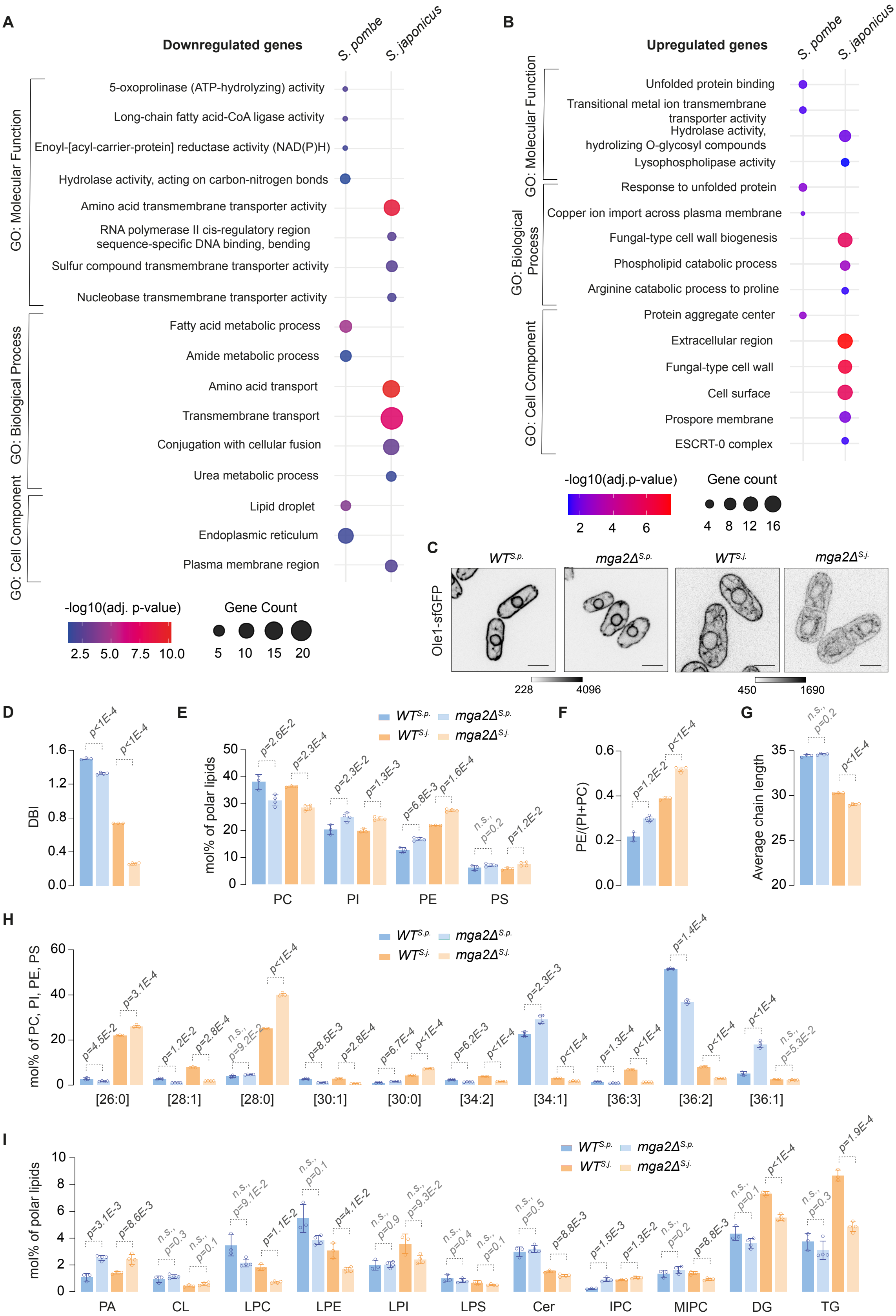

Supplemental Figure S1. The loss of Mga2 results in decreased fatty acid unsaturation and further remodelling of the cellular lipidome in both fission yeasts. (**A**, **B**) Gene Ontology (GO) enrichment analyses of differentially downregulated (**A**) and upregulated (**B**) genes in *mga2Δ* mutant vs wild type in *S. pombe* and *S. japonicus* cells grown in EMM (-0.5 ≥ $\log_2$(fold change) ≥ 0.5 and adjusted p-value < 0.05), performed using g:Profiler. The size of each dot represents the number of terms associated with the GO term, and the colour represents the adjusted p-value. (**C**) Single plane spinning disk confocal images of *S. pombe* and *S. japonicus* wild type (*WT*) and *mga2Δ* cells expressing Ole1-sfGFP grown in YES. Scale bars represent 5 µm. All images were contrasted to the same scale. Greyscale bar is included. (**D**) Comparison of double bond indices (DBI) calculated for the sum of the four main glycerophospholipid (GPL) classes (phosphatidylcholine (PC), phosphatidylinositol (PI), phosphatidylethanolamine (PE) and phosphatidylserine (PS)) in *S. pombe* and *S. japonicus WT* and *mga2Δ* strains grown in EMM. (**E**) Relative abundance of the four main GPL classes in *S. pombe* and *S. japonicus WT* and *mga2Δ* cells grown in EMM. (**F**) Ratio between PE and the sum of PC and PI (PE/(PC+PI)) calculated from the ESI-MS lipidomics data shown in (**E**). (**G**) Average combined FA length calculated for the sum of PC, PI, PE, and PS in *S. pombe* and *S. japonicus WT* and *mga2Δ* strains grown in EMM. (**H**) Molecular species composition calculated for the sum of PC, PI, PE, and PS in *S. pombe* and *S. japonicus WT* and *mga2Δ* cells grown in EMM. The categories are shown as the total number of carbon atoms: total number of double bonds in acyl chains. (**I**) Relative abundance of the indicated lipid classes: phosphatidic acid (PA), cardiolipin (CL), lysophosphatidylcholine (LPC), lysophosphatidylethanolamine (LPE), lysophosphatidylinositol (LPI), lysophosphatidylserine (LPS), ceramide (Cer), inositol phosphoceramide (IPC), mannosyl-inositolphosphoceramide (MIPC), diacylglycerol (DG) and triacylglycerol (TG) in *S. pombe* and *S. japonicus WT* and *mga2Δ* cells grown in EMM. (**D**-**I**) Data are represented as average ± S.D. (n=2 biological, 1 technical repeats for *WT*; 2 biological, 2 technical repeats for *mga2Δ*). p-values are derived from two-tailed unpaired t-test.

(Supplemental Figure S2 overleaf)

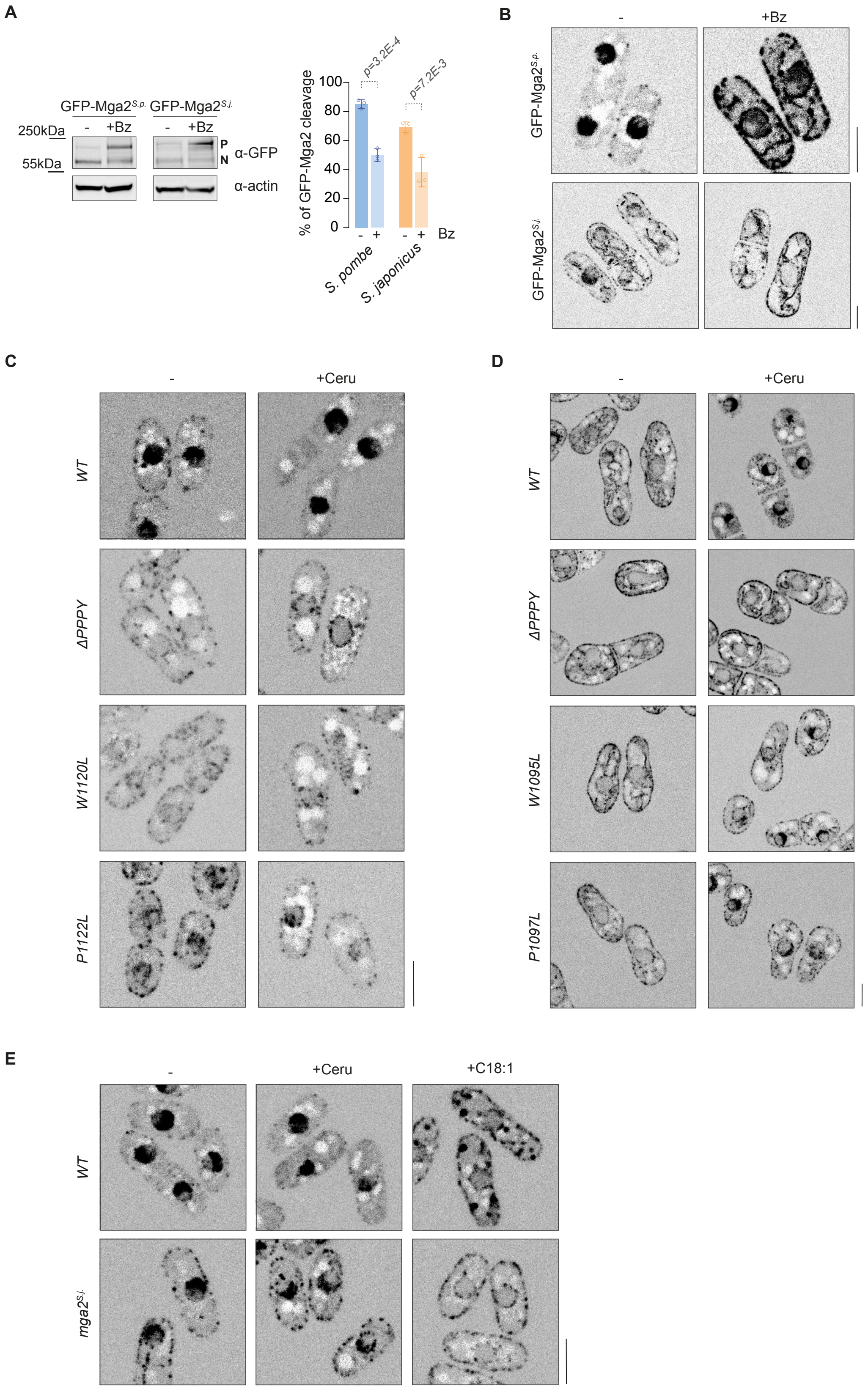

Supplemental Figure S2. Mga2 is proteolytically activated via the proteasome in both sister species. (**A**) Western blot of *S. pombe* and *S. japonicus* cells expressing GFP-Mga2 grown in YES in the absence or the presence of 125 μM of the proteasomal

inhibitor Bortezomib (Bz) for 3.5 hours. Actin was used as a loading control. P and N indicate precursor and cleaved N-terminal transcriptional activator forms, respectively. The right panel shows the quantification of GFP-Mga2 cleavage, calculated as N / (N + P), expressed as percentage. Data are represented as average ± S.D. (n=3 biological repeats). p-values are derived from two-tailed unpaired t-test. (**B**) Single plane spinning disk confocal images of *S. pombe* and *S. japonicus* cells from experiment shown in (A). (**C**, **D**) Single plane spinning disk confocal images of *S. pombe* (**C**) and *S. japonicus* (**D**) cells from experiments shown in Fig. 2E and 2F, respectively. (**E**) Single plane spinning disk confocal images of *S. pombe* cells from experiment shown in Fig. 2G. (**B**-**E**) Scale bars (5 μm) for each species are shown bottom right.

(Supplemental Figure S3 overleaf)

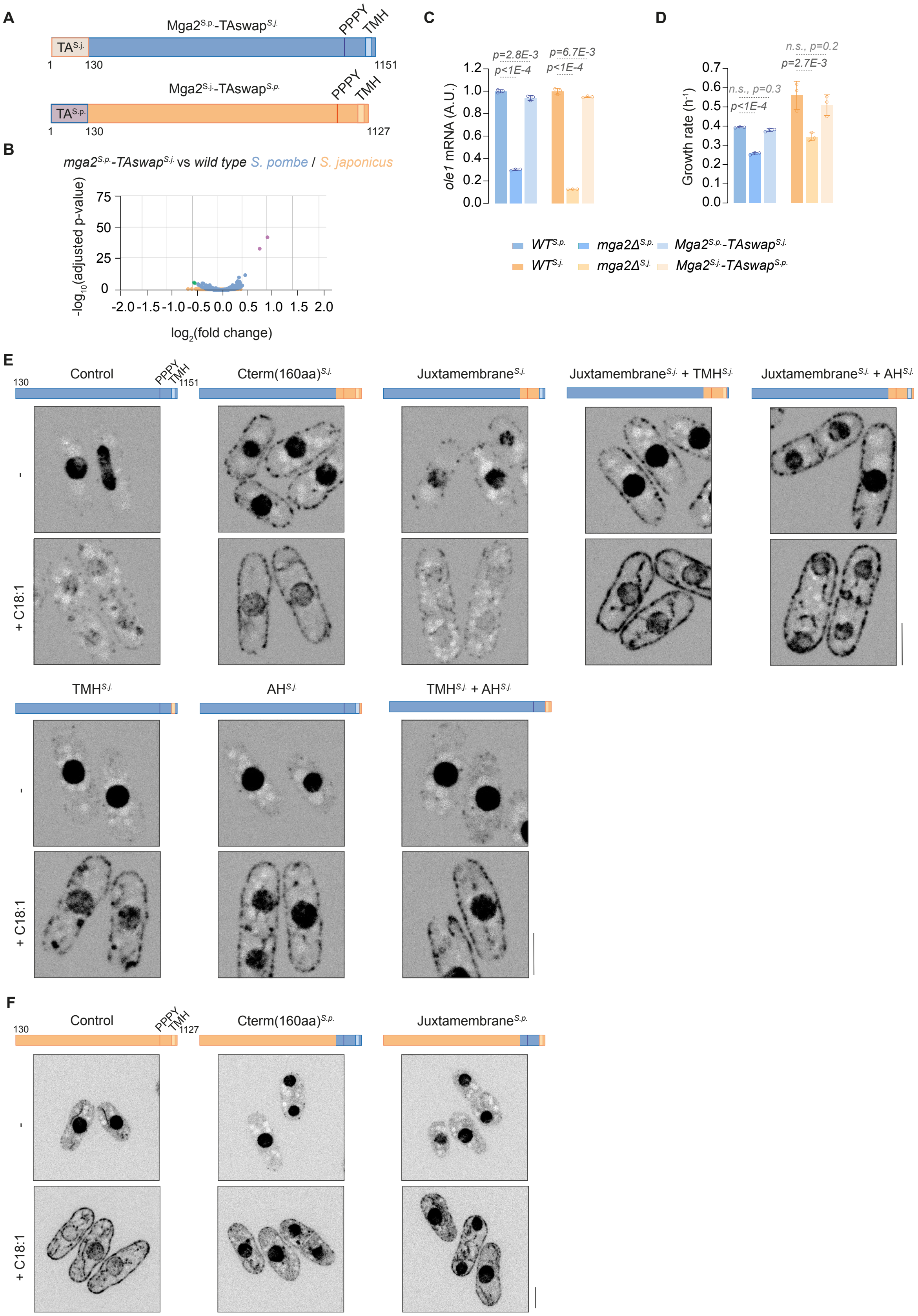

Supplemental Figure S3. Mga2 transcriptional activator domain is functionally interchangeable between *S. pombe* and *S. japonicus*. (**A**) Diagrams showing the Mga2 proteins with transcriptional activator (TA) domain swap. Mga2$^{S.p.}$-TAswap$^{S.j.}$

(top): *S. pombe* Mga2 version with the *S. japonicus* transcriptional activator domain; and Mga2$^{S.j.}$-TAswap$^{S.p.}$ (bottom): *S. japonicus* Mga2 version with the *S. pombe* transcriptional activator domain. The TA, the putative E3 ligase binding site (PPPY) and the predicted transmembrane helix (TMH) are indicated. (**B**) Volcano plot showing RNA-seq data from *S. pombe* (blue) and *S. japonicus* (orange) Mga2-TAswap mutants vs wild type experiments. *S. pombe* genes significantly downregulated (green) or upregulated (magenta) are highlighted ($-0.5 \geq \log_2$(fold change) ≥ 0.5 and adjusted p-value < 0.05). Note that there were no significantly regulated genes in *S. japonicus*. (**C**) Steady state *ole1* mRNA levels in *S. pombe* and *S. japonicus* strains of the indicated genotypes grown in YES, as measured by qPCR, normalized to the wild type (*WT*). (**D**) Comparison of growth rates of *S. pombe* and *S. japonicus* strains of the indicated genotypes grown in YES at 30°C. (**E**) Single plane spinning disk confocal images of *S. pombe* cells from the experiment in Fig. 3c. (**F**) Single plane spinning disk confocal images of *S. japonicus* cells from the experiment in Fig. 3e. (**C**, **D**) Data are represented as average ± S.D. (n=3 biological repeats). p-values are derived from one-way ANOVA followed by multiple comparisons with FDR correction (Benjamini, Krieger and Yekutieli). (**E**, **F**) Scale bars represent 5 µm. Schematics of the corresponding constructs are shown above each image.

(Supplemental Figure S4 overleaf)

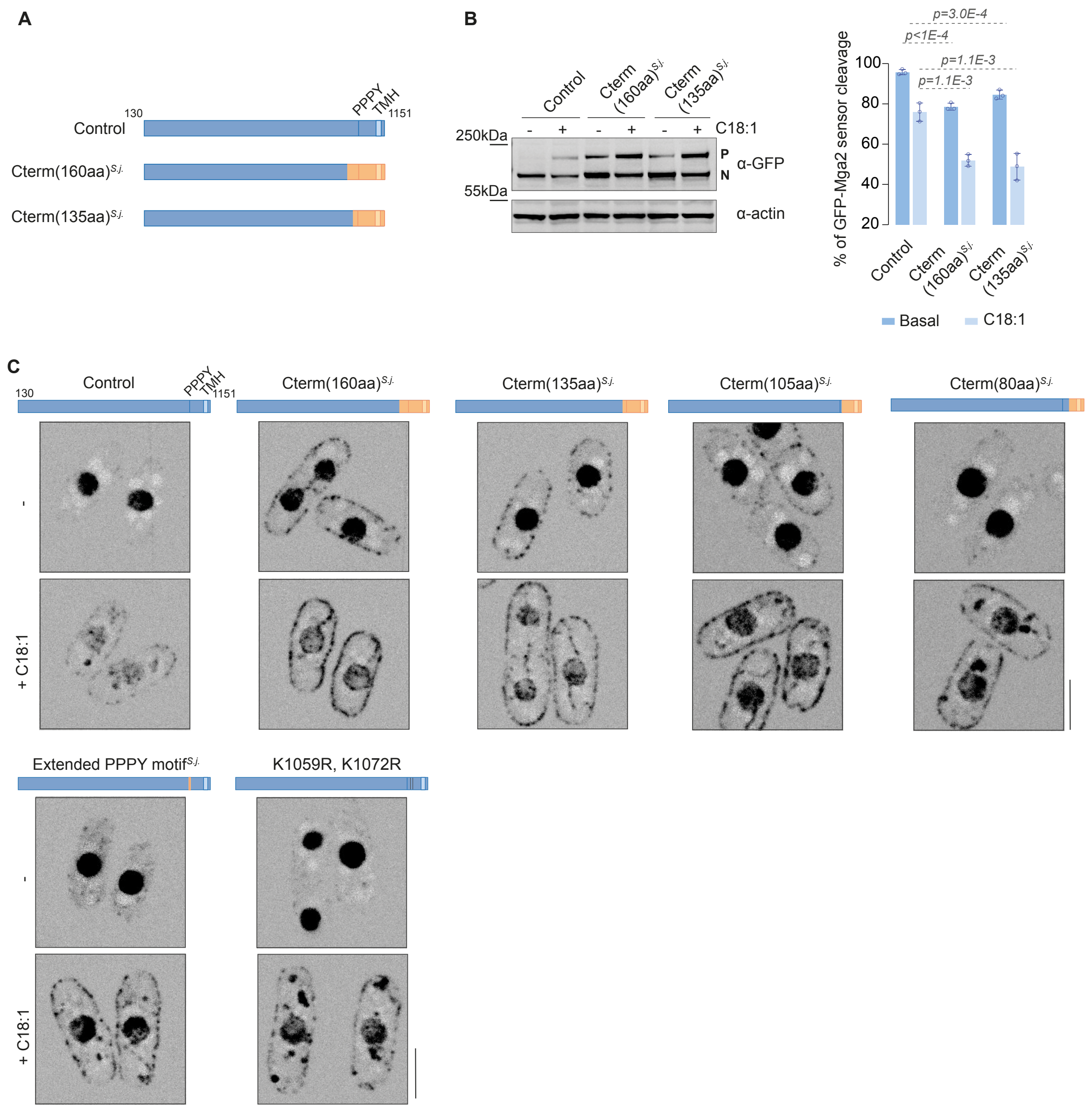

Supplemental Figure S4. Experiments defining the region responsible for the species-specific Mga2 cleavage patterns in fission yeast. (**A**) Schematics of the *S. pombe* chimeric sensor constructs used in experiments shown in (B). (**B**) Western blot of *S. pombe* cells expressing indicated chimeric GFP-Mga2 sensor constructs grown in YES in the absence or the presence of 1 mM oleic acid (C18:1) for 3 hours. Actin was used as a loading control. The right panel shows the quantification of GFP-Mga2 cleavage. P and N indicate precursor and cleaved N-terminal transcriptionally active forms, respectively. Mga2 cleavage is calculated as N / (N + P), expressed as percentage. Data are represented as average ± S.D. (n=3 biological repeats). p-values are derived from one-way ANOVA followed by multiple comparisons with FDR correction (Benjamini, Krieger and Yekutieli). (**C**) Single plane spinning disk confocal images of *S. pombe* cells from experiments shown in Fig. 4C-E and Fig. S4B. Schematics of the corresponding constructs are shown above each image. Scale bars represent 5 μm.

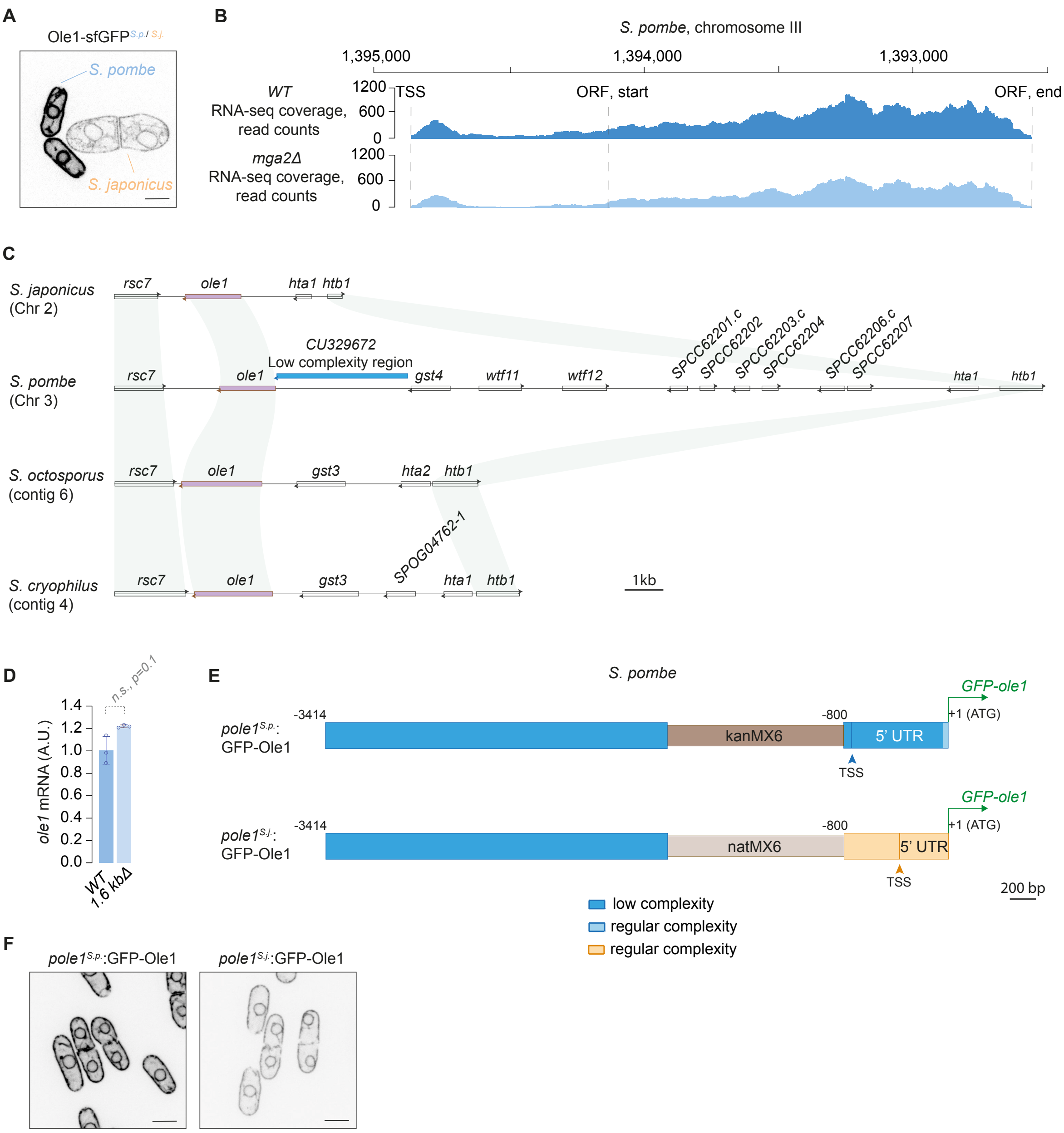

Supplemental Figure S5. *S. pombe ole1* intergenic region shows major rearrangements. (**A**) A single plane spinning disk confocal image of *S. pombe* and *S. japonicus* cells expressing Ole1-sfGFP grown in YES. (**B**) Mapping of *ole1* transcript in *S. pombe* wild type and *mga2Δ* cells, IGV viewer is shown. (**C**) Diagram of the region between *rsc7* and *htb1* in *S. japonicus*, *S. pombe*, *S. octosporus* and *S. cryophilus*. The *ole1* gene is shown in purple. *S. pombe* low complexity region upstream of *ole1* is shown in blue. (**D**) Steady-state *ole1* mRNA levels in *S. pombe* wild type (*WT*) and the mutant lacking 1.6 kb of the low complexity region (*Δ1.6 kb*) grown in YES, as measured by qPCR, normalized to the *WT*. Data are represented as average ± S.D. (n=3 biological repeats). p-values are derived from two-tailed unpaired t-test. (**E**) Schematics showing the *ole1* locus in the *S. pombe* *pole1*$^{S.p.}$ and *pole1*$^{S.j.}$ strains. The *ole1* gene is N-terminally tagged with GFP and expressed from the 800 bp-long promoter regions from *S. pombe* or *S. japonicus*. Promoters are preceded by the antibiotic resistance cassettes integrated in the reverse orientation, and the region

upstream is left unaltered. The location of transcription start sites (TSS) are indicated with arrows. 5' UTR regions extend from the TSS to the start of the ORF. (**F**) Single plane spinning disk confocal images of *pole1*$^{S.p.}$:GFP-Ole1 and *pole1*$^{S.j.}$:GFP-Ole1 *S. pombe* cells, grown in YES. (**A**, **F**) Scale bars represent 5 µm.

Supplemental Files

Supplemental Data 1. Lipidomics. All data presented as Excel tables. (**Table 1**) Detailed lipidomics dataset analysed by shotgun ESI-MS for comparison of *S. pombe* and *S. japonicus wild type* (*WT*) cells at 16°C, 24°C, 32°C or 36°C. Data are expressed as average ± S.D., n= 4 (two biological and two technical repeats). PC, phosphatidylcholine; PE, phosphatidylethanolamine; MMPE, monomethyl PE; DMPE, dimethyl-PE; PI, phosphatidylinositol; PS, phosphatidylserine; PG, phosphatidylglycerol; PA, phosphatidic acid; CL, cardiolipin; LPC, LPE, LPI, LPS, LPA, LCL, the corresponding lyso lipids; Cer, ceramide; IPC, inositolphosphoceramide; MIPC, mannosyl-inositolphosphoceramide; DG, diacylglycerol; TG, triacylglycerol. DBI, double bond index; Av_ChL, average chain length. Sum formulas for glycerophospholipids (GPL) are defined as the lipid class abbreviation followed by the total number of carbons and total number of double bonds for all chains, e.g. PC(34:1). Sum formulas for sphingolipids are defined as the lipid class abbreviation followed by the total number of carbons, total number of double bonds and total number of hydroxyl groups in the long chain base and the fatty acyl moiety, e.g. Cer(44:0:3). Double bond index (DBI) was calculated for GPLs (PC, PE, PI and PS) as Σ(db x [GPLi])/Σ[GPLi], where db is the total number of double bonds in fatty acyls in a given GPL species, and the square bracket indicates mol% of GPLs. Average acyl chain length (Av_ChL) was calculated for GPLs (PC, PE, PI and PS) as Σ(C x [GPLi])/Σ[GPLi], where C is the total number of carbons in fatty acyls in a given GPL, and the square bracket indicates mol% of GPLs. Average species profile was calculated for PC, PE, PI and PS. Please note that the list of lipid species is matched for the two yeast species. Due to the substantial difference in their lipid profile, one can find zero values for certain lipid species in *S. pombe* or *S. japonicus*. (**Table 2**) Principal component analysis for comparison of *S. pombe* and *S. japonicus wild type* (*WT*) cells at 16°C, 24°C, 32°C or 36°C from data in Table 1. (**Table 3**) Detailed lipidomics dataset analysed by shotgun ESI-MS for comparison of *S. pombe* and *S. japonicus WT* and *mga2Δ* cells grown at 30°C. Data are expressed as mol% of polar lipids; polar lipids include all measured lipids except DG and TG. Data are expressed as average ± S.D., (n=two biological, one technical repeats for *WT*; two biological, two technical repeats for *mga2Δ*). DBI, double bond index; Av_ChL, average chain length. Sum formulas for glycerophospholipids (GPL) are defined as the lipid class abbreviation followed by the total number of carbons and total number of double bonds for all chains, e.g. PC(34:1). Sum formulas for sphingolipids are defined as the lipid class abbreviation followed by the total number of carbons, total number of double bonds and total number of hydroxyl groups in the long chain base and the fatty acyl moiety, e.g. Cer(44:0:3). Double bond index (DBI) was calculated for GPLs (PC, PE, PI and PS) as Σ(db x [GPLi])/Σ[GPLi], where db is the total number of double bonds in fatty acyls in a given GPL species, and the square bracket indicates mol% of GPLs. Average acyl chain length (Av_ChL) was calculated for GPLs (PC, PE, PI and PS) as Σ(C x [GPLi])/Σ[GPLi], where C is the total number of carbons in fatty acyls in a given GPL, and the square bracket indicates mol% of GPLs. Average species profile was calculated for PC, PE, PI and PS. Please note that the list of lipid species is matched for the two yeast species. Due to the

substantial difference in their lipid profile, one can find zero values for certain lipid species in *S. pombe* or *S. japonicus*. p-values are derived from two-tailed unpaired t-test; blue fill indicates $p < 0.05$ (**Table 4**) ESI-MS quantification details.

Supplemental Data 2. mRNA-seq. (**Table 1**) DESeq2 analysis results of mRNA sequencing comparing *S. pombe mga2Δ* versus wild type cells grown in EMM at 30°C. n=3 biological repeats. Note that genes are identified by PomBase systematic ID. (**Table 2**) DESeq2 analysis results of mRNA sequencing comparing *S. japonicus mga2Δ* versus wild type cells grown in EMM at 30°C. n=3 biological repeats. Note that genes are identified by JaponicusDB systematic ID. *S. pombe* orthologs are indicated. (**Table 3**) g:Profiler analysis result of Gene Ontology (GO) terms comparing *S. pombe mga2Δ* versus wild type differentially expressed genes (DEGs) from data in Table 1. DEGs were defined as those with an adjusted p-value (Benjamini–Hochberg correction) < 0.05 and an absolute $\log_2$ fold change ≥ 0.5. MF: Molecular Function; BP: Biological Process; CC: Cellular Compartment. Note that genes are identified by PomBase systematic ID. (**Table 4**) g:Profiler analysis result of Gene Ontology (GO) terms comparing *S. japonicus mga2Δ* versus wild type differentially expressed genes (DEGs) from data in Table S2 inferred using *S. pombe* Gene Ontology annotations via orthology mapping. DEGs were defined as those with an adjusted p-value (Benjamini–Hochberg correction) < 0.05 and an absolute $\log_2$ fold change ≥ 0.5. MF: Molecular Function; BP: Biological Process; CC: Cellular Compartment. Note that genes are identified by PomBase systematic IDs for *S. japonicus* orthologs. (**Table 5**) Simple Enrichment Analysis (SEA) results of putative Cbf11-binding motifs in the promoters (1 kb upstream of ORF) of *S. pombe* downregulated genes in *mga2Δ* versus all protein coding genes. (**Table 6**) Simple Enrichment Analysis (SEA) results of putative Cbf11-binding motifs in the promoters (1 kb upstream of ORF) of *S. japonicus* downregulated genes in *mga2Δ* versus all protein coding genes.

Supplemental Data 3. *S. pombe* Mga2 variants. (**Table 1**) List of single nucleotide variants located in *mga2* across 57 *S. pombe* natural isolates, along with their predicted effects.

Supplemental Data 4. List of fission yeast strains, primers and plasmids used in this study. (**Table 1**) *S. pombe* and *S. japonicus* strains used in this study are listed in order of appearance. (**Table 2**) Primers used to generate deletions, fluorescent protein tagging and replacements, genotyping primers and RT-qPCR primers are listed. (**Table 3**) Plasmids generated to obtain chimeric constructs are listed. (**Table 4**) The correct sequence of *S. japonicus* Mga2 protein is indicated.
